# Prevalence and phenotypic characterization of *Salmonella enterica* isolates from three species of wild marine turtles in Grenada, West Indies

**DOI:** 10.14202/vetworld.2021.222-229

**Published:** 2021-01-25

**Authors:** Jonnel J. Edwards, Victor A. Amadi, Esteban Soto, Michele T. Jay-Russel, Peiman Aminabadi, Kirsten Kenelty, Kate Charles, Gitanjali Arya, Ketna Mistry, Roxanne Nicholas, Brian P. Butler, David Marancik

**Affiliations:** 1 Department of Pathobiology, School of Veterinary Medicine, St. George’s University, Grenada, West Indies; 2 Department of Pathobiology, School of Veterinary Medicine, University of California-Davis, Davis, California, USA; 3 Western Center for Food Safety, University of California, Davis, California, USA; 4 Department of Medicine and Epidemiology, School of Veterinary Medicine, University of California, Davis, California, USA; 5 Ocean Spirits Inc., Grenada, West Indies; 6 Office of International des Epizooties Salmonella Reference Laboratory, National Microbiology Laboratory, Public Health Agency of Canada, Guelph, Ontario, Canada

**Keywords:** antimicrobials, marine turtles, pulsotypes, *Salmonella enterica*, serotypes, zoonosis

## Abstract

**Background and Aim:**

*Salmonella enterica* causes enteric disease in mammals and may potentially be transmitted from marine turtles that shed the pathogen in the environment. Marine turtle-associated human salmonellosis is a potential public health concern in Grenada, as the island supports populations of leatherback turtles (*Dermochelys coriacea*), hawksbill turtles (*Eretmochelys imbricata*), and green turtles (*Chelonia mydas*) that interface with veterinarians and conservation workers, the local population, and the thousands of visitors that frequent the island yearly. To date, the prevalence of *S. enterica* has only been examined in a small subset of marine turtles in the Caribbean and no studies have been conducted in Grenada. The aim of this study was to quantify the prevalence of *S. enterica* in leatherback, hawksbill and green turtles in Grenada, characterize phenotypes and DNA profiles, and explore the potential risk to human health in the region.

**Materials and Methods:**

A total of 102 cloacal swabs were obtained from nesting leatherback turtles and foraging hawksbill and green turtles. Samples were cultured on enrichment and selective media and isolates were phenotypically characterized using serotyping, pulsed-phase gel electrophoresis, and antibiotic susceptibility. Enrichment broths were additionally screened by polymerase chain reaction (PCR) using *S. enterica*-specific primers.

**Results:**

*S. enterica* was cultured from 15/57 (26.3%) leatherback turtles, 0/28 hawksbill, and 0/17 green turtles. This included *S*. *enterica* serovars Montevideo, *S*. I:4,5,12:i:-, *Salmonella* Typhimurium, *Salmonella* Newport, *S*. I:6,7:-:-, and *S*. I:4,5,12:-:-. Five/15 leatherback turtles carried multiple serovars. Eight pulsotype groups were identified with multiple clustering; however, there was no clear association between pulsotype group and serotype profile. Five/71 isolates showed resistance to streptomycin or ampicillin. Twenty-one/57 leatherback turtles, 14/28 hawksbill turtles, and 8/17 green turtles tested positive for *S. enterica* by quantitative PCR.

**Conclusion:**

Nesting leatherback turtles actively shed *S. enterica* and poses a risk for zoonosis; however, the presence of viable pathogen in green and hawksbill species is unclear. These findings help elucidate the role of marine turtles as potential sources of zoonotic *S. enterica* and provide baseline data for one health research in Grenada and the wider Caribbean region.

## Introduction

*Salmonella enterica* is a Gram-negative, facultative anaerobic bacterium that causes an estimated 3.8 million reported illnesses and 155,000 deaths per year worldwide [1]. There are over 2,500 serotypes, some of which are zoonotic and have shown varying levels of pathogenicity [2]. *S. enterica* lives as normal flora in the intestinal tract of some vertebrates and is subsequently shed into the environment by fecal contamination where it can survive for long periods of time outside the host [3]. Human exposure occurs through direct contact with carrier animals [4], or by ingestion of contaminated food such as eggs, vegetables [5,6], and raw or undercooked meats [7].

*S. enterica* is the most commonly isolated human enteropathogen in Grenada [8] and the greater Caribbean region [9]. Despite an estimated 69% of enteric illnesses being unreported, Grenada incurs an annual cost of $703,950 USD associated with treatment of acute gastroenteritis [8]. This data suggest that the health impacts and financial cost from this disease are substantial. Grenada would benefit from having a better understanding of the exposure risks of Salmonella-associated infectious gastroenteritis on the island as well as the potential for antibiotic resistance [10]. The risk of zoonosis is greater in developing countries due to the close proximity between agriculture and communities [11]. In Grenada, farming and fishing practices are established within communities which may increase human exposure to animal reservoirs and potentially contaminated soil or sand. *S. enterica* has been isolated from various terrestrial animals on the island such as cane toads (*Bufo marinus*) [12], Indian mongooses (*Herpestes auropunctatus*) [13], blue land crabs (*Cardisoma guanhumi*) [14], and iguanas (*Iguana iguana*) [15]. Among these studies, the most commonly isolated serotypes were *S. enterica* serovar Montevideo, *Salmonella* Rubislaw, *Salmonella* Newport, *Salmonella* Javiana, and *Salmonella* Oranienburg – all serotypes previously described as potentially zoonotic [16-19]. Sporadic resistance to tetracycline, streptomycin, amoxicillin, and ampicillin were observed [12,13]. Marine turtles may also represent a potential source of zoonosis for *S. enterica* in Grenada. Consumption of green turtle (*Chelonia mydas*) meat has been linked with disease outbreaks of *Salmonella* Muenchen [20] and *Salmonella* Chester [21] in Australia. An uncharacterized *S. enterica* isolate has been cultured from leatherback turtle eggs in Grenada [22], which is historically consumed by the local population. In the Caribbean island of St. Kitts, *S. enterica* was cultured from the cloaca of 3/21 [23] and 2/9 [24] leatherback turtles while 14 hawksbill turtles (*Eretmochelys imbricata*) and nine green turtles were culture-negative [24]. Among the obtained isolates in leatherback turtles, serotypes Montevideo and Newport were most prevalent indicating potential zoonotic risks. Grenada supports similar, although larger, population of marine turtles including nesting leatherback turtles as well as foraging and nesting populations of hawksbill and green turtles. These species interface with local and tourist populations and may pose a risk of zoonosis in three ways: (1) Consumption of contaminated turtle meat and eggs during the legal hunting season [25], (2) handling of turtles and eggs by conservation and veterinary personnel on the island, and (3) fecal contamination of sand during the 5 months nesting season and inshore waters year-round, posing a potential risk to those who utilize these areas for fishing, ecotourism, or recreation.

The goal of this study was to quantify the prevalence of *S. enterica* in leatherback, hawksbill and green turtles in Grenada, characterize phenotypes and DNA profiles, and explore the potential risk to human health in the region. As there are limited reports on the prevalence and characteristics of *S. enterica* in marine turtles in the Caribbean region, data from this study will further establish the role of a statistically significant population of marine turtles in the dissemination of the pathogen in the marine environment.

## Materials and Methods

### Ethical approval

The protocol and procedures of this study were approved by the St. George’s University Institutional Animal Care and Use Committee (IACUC-16017-R) and approval and permitting was obtained from the Grenada Ministry of Agriculture, Forestry, Lands, and Fisheries.

### Sampling, study period, and location

**Figure-1:**
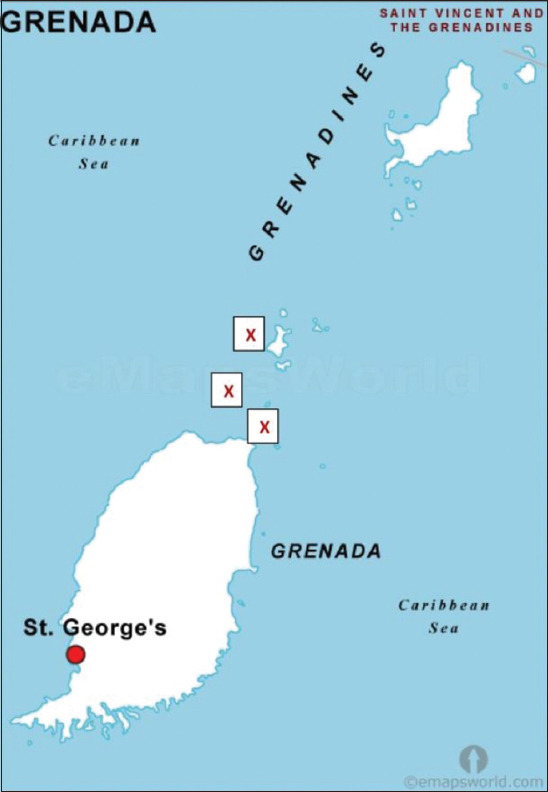
Map of Grenada showing sample collection sites (X). [Source: https://emapsworld.com/grenada-capital-map.html, pasted onto image by Jonnel Edward].

Cloacal cultures were obtained from nesting leatherback turtles from April to August 2017 on Levera Beach, Grenada (Figure-1). Samples were obtained under long-wavelength light and immediately following oviposition, defined by the expulsion of yolkless eggs, to avoid disrupting the nesting process. Foraging hawksbill and green turtles were sampled in this same region offshore during the day (Figure-1). Turtles were hand-caught by free-divers and taken aboard a boat within 30 s of capture. To obtain cloacal samples, the tail was gently lifted to identify the cloaca and a sterile culturette (Thermo Scientific Remel BactiSwab, USA) was inserted approximately 5 cm into the cloaca and spun 6 times [23]. Culturettes were placed on ice for field storage until transfer to St. George’s University, School of Veterinary Medicine for bacterial culture within 24 h of sample collection.

### Isolation and identification of S. enterica

Each cloacal swab was transferred to 10 mL trypticase soy enrichment broth (TSB) (BD Bacto, Sparks, MD, USA) and incubated at 37°C for 24 h in static conditions. Following incubation, 100 μL of each sample in TSB was aliquoted into 10 mL Rappaport-Vassiliadis enrichment broth (Oxoid, Hants, UK) and incubated at 47°C for 48 h in static conditions. The remainder of samples in TSB was stored at −80° for quantitative polymerase chain reaction (qPCR). Following incubation, 100 μL of each sample was streaked onto one plate of xylose lysine deoxycholate (XLD, CA, USA) agar (Criterion dehydrated culture media) which is selective for *Salmonella* spp. [26] and incubated at 37°C for 24 h in aerobic conditions. To explore the presence of mixed-serovar infections within one sample, 1-5 individual colonies per plate with typical *Salmonella* spp. morphology (colonies with a black center) were re-streaked onto XLD agar, and incubated at 37°C for 24 h [27]. Single colonies from the XLD agar were sub-cultured on trypticase soy agar plates (BD Difco, Sparks, MD, USA) at 37°C for 24 h. Resulting individual colonies were tested for agglutination using *Salmonella* O antiserum poly A-I and Vi (BD Difco™). Agglutination-positive cultures were inoculated onto analytical profile index strips (API-20E^®^) for preliminary identification of *Salmonella* spp. Reference strain *S. enterica* subspecies enterica ATCC13311 was used as a positive control [28]. Identified pure *Salmonella* cultures were stored in 10% sterile skim milk solution at −80°C.

DNA was isolated from 1.5 mL TSB broth using the DNEasy Blood and Tissue Kit (QUIAGEN, Hilden, Germany) following manufacturer’s protocol. As a minor modification to eliminate any inhibitors contained in TSB, 1.5 mL of broth was centrifuged for 5 min and pellets were washed with 0.2 mL phosphate-buffered saline before carrying out the kit protocol. DNA products were nanodropped and normalized to ~50 ng/μL DNA concentration, and run in triplicate qPCR reaction using the invA probe (FAM-CGTCACCTTTGATAAACTTCATCGCA–BHQ1) and primer sets (forward AGCGTACTGGAAAGGGAAAG; reverse 3’-5’ ATACCGCCAATAAAGTTCACAAAG) [29].

Serotyping [30,31] was performed on all cultured isolates by the World Organization for Animal Health (Office International des Epizooties; OIE) Salmonella Reference Laboratory of the Public Health Agency of Canada’s National Microbiology Laboratory at Guelph, Ontario, Canada [32]. Serovars were named based on an established antigenic formula [33].

Pulsotyping was performed on whole bacterial cultures of 15 randomly selected isolates using pulse-field gel electrophoresis (PFGE) to examine the DNA fingerprint and genetic clonality of *Salmonella* isolates obtained from leatherback cloacal swabs [34].

### Antimicrobial sensitivity of S. enterica isolates

Antimicrobial sensitivity was determined using the standard Kirby–Bauer disk diffusion method on Mueller–Hinton agar (Remel, KS, USA), and zones of inhibition were measured as recommended by the Clinical and Laboratory Standards Institute [35] using *S. enterica* subspecies enterica ATCC13311 as a reference strain. *Salmonella* isolates were tested against the following drugs: Cefotaxime (30 μg), ceftazidime (30 μg), nalidixic acid (30 μg), ciprofloxacin (5 μg), streptomycin (10 μg), amoxicillin-clavulanic acid (30 μg), ampicillin (30 μg), enrofloxacin (5 μg), gentamicin (10 μg), sulfamethoxazole (23.75 μg), and tetracycline (30 μg) (BD Difco, Sparks, MD, USA). These antimicrobials were selected based on their common use for treatment of Salmonellosis in Grenada [27].

### Statistical analysis

Prevalence level of *S. enterica* by culture and qPCR was compared using an unpaired t-test and prevalence levels were compared between leatherback, hawksbill, and green turtles using a one-way analysis of variance. All statistics were run with a significance level of p<0.05.

## Results

**Figure-2:**
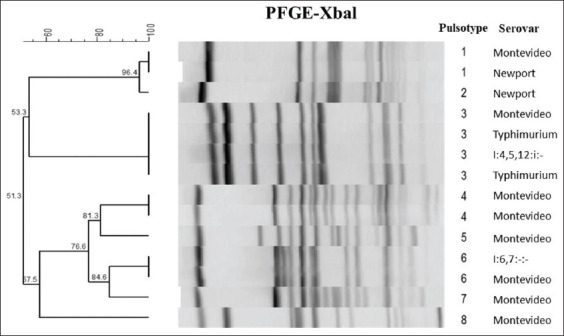
Pulsotype group and associated serotype of 15 randomly selected Salmonella enterica isolates.

*S. enterica* was cultured from cloacal swabs from 15/57 (26.3%) nesting leatherback turtles, 0/28 foraging hawksbill turtles, and 0/17 foraging green turtles (Table-1). When examined by quantitative PCR, prevalence increased to 21/57 (36.8%) in leatherback turtles, 14/28 (50%) in hawksbill turtles, and 8/17 (47%) in green turtles (Table-1). *Salmonella* Montevideo was the most commonly cultured serotype, followed by *S*. I:4,5,12:i:-, *Salmonella* Typhimurium, *S*. Newport, *S*. I:6,7:-:-, and *S*. I:4,5,12:-:-, respectively. Five/15 (33.3%) turtles carried multiple serovars (Table-2). Fifteen *S. enterica* isolates examined by PFGE comprised eight distinct pulsotypes with multiple clustering between groups and no clear association between pulsotype groups and serotype profiles (Figure-2). Five/71 (7.1%) isolates showed resistance to streptomycin or ampicillin.

**Table-1:** Prevalence of *Salmonella enterica* in the cloaca of leatherback, hawksbill, and green turtles using bacterial culture and qPCR.

Turtle species	n	Number of positive turtles by bacterial culture (%)	Number of positive turtles by qPCR (%)
Leatherback	57	15 (26.3)	21 (36.8)
Hawksbill	28	0	14 (50.0)
Green	17	0	8 (47.0)

qPCR=Quantitative polymerase chain reaction

**Table-2:** Mixed *Salmonella enterica* serovars isolated from single cloacal cultures from leatherback turtles.

Leatherback sample no.	Number of serotypes isolated	Serotypes identified
L14	2	*S.* Montevideo, *S.* I:6,7:-:-
L36	3	*S.* Montevideo, *S.* Typhimurium, *S*. I:4,5,12:i:-
L37	2	*S*. Montevideo, *S*. I:4,5,12:i:-
L40	2	*S.* Typhimurium, *S*. I:4,5,12:i:-
L45	4	*S.* Montevideo, *S.* Typhimurium, *S*. I:4,5,12:i:-, *S*. I:4,5,12:-:-

*S=Salmonella*, *S.* Montevideo=*Salmonella* Montevideo, *S.* Typhimurium=*Salmonella* Typhimurium

## Discussion

The goal of this study was to isolate and phenotypically characterize *S. enterica* from cloacal samples of leatherback, hawksbill, and green turtles found in Grenada and to compare prevalence among each turtle species. Overall, the prevalence of *S*. *enterica* was higher in leatherback turtles compared with hawksbill and green turtles by bacterial culture; however, there was no statistically significant difference in prevalence among the three species when examined by qPCR. Serovars *S*. I:4,5,12:i:-, *S*. I:4,5,12:-:- and *S*. I:6,7:-:- were isolated for the first time from leatherback turtles and eight distinct pulsotype groups were characterized and comprised isolates with varying serotypes. Results from this study help elucidate the role of marine turtles as carriers of *S. enterica* and the zoonotic risk they pose when they interface with humans.

The 24% prevalence of *S*. *enterica* in nesting leatherback turtles by culture was similar to the 14% [23] recovery found in nesting leatherback turtles in St. Kitts. In addition, similar to results described in this study, foraging hawksbill and green turtles were also culture-negative in St. Kitts. When examined by qPCR, however, *S. enterica* was detected in 7% and 0% of hawksbill and green turtles in St. Kitts, respectively [24], which is considerably lower than the 50% and 47% prevalence demonstrated, respectively, in hawksbill and green turtles in Grenada. This disparity in prevalence by molecular methods may be attributed to the larger sample size, difference in specificity of primer sets, or it may represent an epidemiologic difference between regions of the Caribbean.

Recovery of viable *S. enterica* from leatherback turtles but not hawksbill or green turtles suggests that leatherback turtles were more actively shedding the bacteria at the time of sampling. The circumstances associated with this finding are unknown but may be multifactorial. As logistical and size constraints limit accessibility to foraging leatherback turtles, samples were obtained while leatherback turtles nested on the beach. Samples from hawksbill and green turtles were obtained while they foraged in open coastal waters as they do not typically nest on the mainland of Grenada. This limitation naturally biased the sample population to include sexually mature, female leatherback turtles and a mix of male/female and sexually mature/immature hawksbill and green turtles. In addition to age and gender differences, the act of nesting may also affect culture results. It is possible that egg laying increases viable *S. enterica* within the cloacal cavity during oviposition as transmission of *Salmonella* spp. to the egg has been described in leatherback turtles [22]. Immunosuppression has been shown to increase proliferation and shedding of bacteria, including *S. enterica*, in mammals and other cold-blooded species [36-38]. Although the effect of nesting on the immune response in sea turtles is relatively uncharacterized, leatherback turtles may potentially be stressed during nesting and therefore shed the pathogen more actively than non-nesting hawksbill and green turtles. Nesting leatherback turtles had contact with the sand while foraging hard-shell turtles did not, especially male and juvenile turtles as only mature females return to land once hatched. The effect that sand contact may have on dissemination of *S. enterica* in turtles is unknown. *S. enterica* has been detected by PCR in sand within leatherback turtle nests [24]. It is possible that bacterial contamination of the sand could result in transmission of *S. enterica* to nesting turtles that subsequently come ashore to nest. This may be further explored by examining the presence of *S. enterica* from sand in nests in Grenada and by sampling nesting hard-shell turtles, or if logistically feasible, sampling foraging leatherback turtles.

*Salmonella* Montevideo was the most commonly isolated serovar as it was found in two-third of isolates from culture-positive leatherback turtles. This is similar to findings in St. Kitts [23] and this relatively high prevalence distinguishes *S*. Montevideo as a public health risk for salmonellosis from marine turtles. This serovar is known to cause enteric disease outbreaks in humans [39,40] and has been described as environmentally robust due to its ability to form biofilms [41,42] and acts as a long-term environmental contaminant [43]. The presence of *S*. Montevideo in both marine and terrestrial cold-blooded and warm-blooded vertebrates as well as invertebrates [12-14] highlights its potential zoonotic risk at the wildlife-human interface in Grenada. Subsequent research into the genetic relatedness of isolates from these different species may provide further understanding of host-bacterial interactions and epidemiology of *S*. *enterica* on the island.

*S*. Typhimurium, *S*. I:4,5,12:i:-, and *S*. Newport were also relatively common at 20-30% prevalence in culture-positive turtles and all have been associated with salmonellosis [44]. *S*. Typhimurium has been described as a multidrug-resistant, invasive serovar with varying degrees of host adaptation [45] and can cause aggressive gastroenteritis in humans [46]. Multidrug resistance was not observed in *S*. Typhimurium isolates in this study. *Salmonella* I:4,5,12:i:- is a monophasic variant of *S*. Typhimurium (antigenic formula 4, [5], 12:i:1,2). It has been described to be genetically closely related to *S*. Typhimurium [47,48] with a similar pathogenicity gene repertoire, although phenotypic differences in motile capacity and cell adhesion have been described [49]. *Salmonella* I:4,5,12:i:- has been described as a potential health risk to humans based on transmission from farmed pigs during processing [48]. *S*. Newport, previously described as zoonotic, appears to be relatively prevalent in leatherback turtles [24] and green iguanas [15]. *Salmonella* I:6,7:-:- and *S*. I:4,5,12:-:-, which were each isolated from one leatherback turtle, have not been well characterized and there are no reports of human infection in the literature to indicate their pathogenicity or potential zoonotic risk.

There were five instances where multiple *Salmonella* serovars were isolated from the same cloacal sample. This included two turtles that carried *S*. Montevideo, *S*. Typhimurium, and *S*. I:4,5,12:i:- and one turtle each that carried *S*. Montevideo and *S*. I:4,5,12:i:-, *S*. Typhimurium and *S*. I:4,5,12:i:-, and *S*. Montevideo and *S*. I:6,7:-:-. To the best of our knowledge, this is the first description of multi-serovar infections in marine turtles. Multi-serovar carriers have been described in other species including iguanas and mongooses in Grenada [13,15] and the combination of serovars that were isolated from these terrestrial species was not similar to those found in leatherback turtles. The carriage of multiple *Salmonella* serovars indicates the potential for leatherback turtles to transfer mixed infections to humans which has been previously described from terrestrial food animals [50,51]. Multi-serovar infection in farmed animals and retail meats has been associated with transfer of antibiotic resistance genes between isolates [52,53]. Although exposure of marine turtles to antibiotics and development of drug-resistant bacteria is likely minimal, the risk of agricultural run-off in the dissemination of multidrug-resistant *S. enterica* isolates to marine turtles warrants investigation.

There was no clear relationship between serovar profiles and pulsotype groups for the 15 *S. enterica* isolates examined. For example, eight *S*. Montevideo isolates were spread between seven different pulsotype groups and often clustered more closely with isolates from other serovars rather than with each other. This included pulsotype Group 3 that was comprised *S*. Montevideo, two *S*. Typhimurium isolates, and *S*. I:4,5,12:i:-. Similar findings have been described in *S. enterica* isolates from poultry [54], indicating that within this bacterial species there is little association between expressed surface antigens and large DNA genotypic profiles. The advantage of pulsotyping in contrast to serotyping to establish epidemiology is unknown, and the association of pulsotype groups with pathogenicity or host-type is unclear. Therefore, it may be pertinent to conduct pulsotyping of *S. enterica* isolates archived from previous studies of terrestrial species in Grenada. This may better establish genetic clonality among isolates between species and help elucidate the role of these animals in the epidemiology of salmonellosis.

Isolates were largely sensitive to the antimicrobials tested, with sporadic evidence of resistance. The most prevalent resistance was to streptomycin in 7% of isolates. Studies of green iguanas and Indian mongooses in Grenada have shown intermediate resistance to streptomycin [13,15]. Similar findings have also been published outside the Caribbean region, where resistance to streptomycin was observed in *S. enterica* isolates cultured from slaughtered bovines and ovines in Ethiopia [55]. The varying levels of antimicrobial sensitivity to streptomycin may potentially impact the use of this drug to treat salmonellosis.

Thirty-seven/102 (36.2%) turtles that were culture-negative were interpreted as positive by qPCR. This result was anticipated based on the relatively higher sensitivity of the qPCR assay. Although *S. enterica* may have been present in the cloaca, it may not have proliferated on the media used, at the specific temperature and incubation period, or it may have been outcompeted by less fastidious bacteria. In addition, not all *Salmonella* spp. strains are hydrogen sulfide-positive [56] and therefore some positive samples may have been missed due to selection of colonies based on this particular phenotype. These results indicate the importance of using multiple detection methods for the surveillance of various strains of *S. enterica* from animals.

This research has compiled statistically significant data on the prevalence and characteristics of *S. enterica* in marine turtles in the region. There are no available data regarding phenotypic or genotypic characteristics of human isolates of *S. enterica* in Grenada, and therefore we cannot determine at this time that marine turtles are a confirmed source of human salmonellosis on the island. However, due to the interface that humans in Grenada share with marine turtles, it is reasonable to suggest that these reptiles are a potential source of zoonotic transmission. Further sampling in marine turtles, sand, and water may provide additional epidemiologic data to elucidate the role of these species in spreading *S. enterica* in the environment. This information also will drive educational guidelines to reduce the risk of salmonellosis in fisherman, veterinarians, ecotourists, conservation workers, and the general public who interface with these species.

## Conclusion

These results demonstrate that nesting leatherback turtles actively shed *S. enterica* and pose a risk for zoonosis; however, the presence of viable pathogen in green and hawksbill species warrants examination. Baseline data from this study will propel further investigation of the zoonotic potential and public health implications of *S*. *enterica* carriage in marine turtles in the region.

## Authors’ Contributions

JJE contributed to study design, sample collection and processing, assay development, data analysis, and manuscript preparation. VAA contributed to study design, sample processing, assay development, data analysis, and manuscript preparation. ES contributed to sample processing, assay development, and manuscript preparation. MTJ and PA contributed to PFGE assay development and sample processing. KK contributed to qPCR assay development and sample processing. KC participated in sample collection. GA and KM contributed to assay development and sample processing. RN participated in sample processing. BPB contributed to study design, sample collection, and manuscript preparation. DM contributed to study design, sample collection, data analysis, and manuscript preparation. All authors read and approved the final manuscript.
